# Current knowledge on the genetic background of developmental dysplasia of the hip and the histomorphological status of the cartilage

**DOI:** 10.3325/cmj.2020.61.260

**Published:** 2020-06

**Authors:** Ivan Bohaček, Mihovil Plečko, Tea Duvančić, Tomislav Smoljanović, Andreja Vukasović Barišić, Domagoj Delimar

**Affiliations:** 1 Department of Orthopedic Surgery, University Hospital Center Zagreb, University of Zagreb School of Medicine, Zagreb, Croatia; 2 University of Zagreb School of Medicine, Zagreb, Croatia; 3 General Hospital Bjelovar, Bjelovar, Croatia

## Abstract

Developmental dysplasia of the hip (DDH) represents a morphological abnormality characterized by the incongruity of femoral head and acetabulum. It ranges from mild dysplastic changes to complete dislocation. DDH has been associated with several hereditary and environmental risk factors, which could explain the incidence variability among different countries. Numerous genes may be involved in the disease etiology and progression. However, there are controversies in the literature regarding some of these genes. DDH-induced secondary osteoarthritis (OA) is characterized by changes in the macromolecule content of the cartilage and the expression of cartilage degradation markers. In addition, it exhibits a pattern of specific histological changes, with several reported differences between primary and DDH-induced secondary OA. The articular cartilage of patients with DDH shows specific radiological characteristics, including changes visible already in infancy, but also at pre-arthritic stages, early stages of OA, and in fully developed DDH-induced secondary OA. Although DDH has been extensively researched in different disease stages, the etiology of the disorder still remains uncertain. This review focuses on the current knowledge on the histomorphological status of the cartilage and the genetic background of DDH.

Developmental dysplasia of the hip (DDH) is a pathological condition characterized by the incongruity of the femoral head and acetabulum. It encompasses a wide range of conditions, from mild dysplastic changes to complete dislocation (1). There is still no universally agreed definition of DDH, but the most frequently used ones are the anatomic definition, which defines DDH as abnormal development of the articulating bones of the hip, and radiological definition, which defines DDH as a structural abnormality of the acetabulum or femoral head with continuous Shenton’s line. In the same manner, subluxation is defined as structural abnormalities and discontinuation of Shenton’s line, and luxation as an abnormality without contact between the femur and acetabulum (2). The hip joint space forms in the 7th-8th week of gestation by autolytic degeneration, and the hip joint basic morphology is developed by the end of week 10. Only after this point can dislocation occur (3). In DDH, the acetabulum is altered both in form and orientation, with physiological width but with increased length and decreased depth, thus insufficiently covering the femoral head. The femoral head is usually aspherical and has increased anteversion, together with a coxa valga deformity. The insufficient coverage over the femoral head, the hip center of rotation lateralization, and a smaller contact area may lead to asymmetric dispersion of forces in the hip. This results in increased mechanical stress on the cartilage beyond the level of physiological tolerance and leads to the development of cartilage lesions and secondary osteoarthritis (OA) (4,5). Patients with untreated DDH may often require total hip replacement, for which many efficient approaches have been described (6-10).

Several hereditary and environmental factors have been associated with DDH, and these may explain the incidence variability among different countries (11). The risk factors for DDH include being firstborn, female sex, breech birth, presence of oligohydramnios, positive family history, joint laxity, swaddling, etc (12). According to Barlow (13), 1 in 60 newborns have hip instability, and more than 60% of them stabilize in the first week without any treatment, while 88% stabilize within the first two months. The remaining 12% have DDH that will persist without therapy. The risk for a child to develop DDH is 6% if one of his or her siblings has DDH, 12% if one of his or her parents has DDH, and 36% if both a parent and a sibling have DDH (14). Canadian indigenous people who in their childhood were swaddled on a cradleboard had 10 times higher incidence of DDH (15). The etiology of DDH still remains uncertain, which is why we reviewed the current knowledge on the histomorphological status of the cartilage and on the genetic background of DDH.

## GENETICS AND TRANSCRIPTOMICS

There are two groups of patients with DDH: a) the group with acetabular dysplasia caused by a large number of gene variations, usually diagnosed later in life, and b) the group with generalized soft tissue and joint laxity, usually diagnosed in the neonatal period, when hip dislocation occurs (14). Each gene involved in DDH pathophysiology has a modest effect, thus making the identification of such genes a challenge (16). This may explain the contradictory results of genetic association-based studies of DDH (17). Studies identified several genes that may contribute to the DDH etiology (Table 1) (18-32). However, other authors showed no association between certain single nucleotide polymorphisms or genes and DDH (Table 2) (33-36). These contradictory results may be explained by the population-specificity of the detected genes and single nucleotide polymorphisms. Therefore, these results should not be used as a basis for causal inference and indicate the need for thorough population-specific studies.

**Table 1:** Studies of genetic background of developmental dysplasia of the hip (DDH) showing correlation between genes and the disease in different populations*

Locus	Gene	Protein	Function	Population	Correlation to DDH	Reference
chromosome 1q24	dermatopontin *(DPT)*	dermatopontin	role in angiogenesis, fetal development, wound healing, tumor metastasis; increased expression in MSCs in chondrogenic differentiation	Japanese	overexpressed in DDH OA	(18)
chromosome 1q32	ATPase plasma membrane Ca^2+^ transporting 4 (*ATP2B4)*	ATPase 2B4	regulation of bone homeostasis	Saudi Arabian	interaction between rare heterozygous variants of *HSPG2* and *ATP2B4* genes is connected to DDH in a studied family due to a combined effect of both variants; one member was asymptomatic, which suggests incomplete penetration of this variants	(19)
chromosome 1p36	heparan sulfate proteoglycan 2 (*HSPG2)*	perlecan	proteoglycan that cross-links extracellular matrix components and cell-surface molecules important in musculoskeletal development			
chromosome 4q12	insulin-like growth factor-binding protein 7 (*IGFBP7)*	insulin-like growth factor-binding protein 7	development of cartilage, muscles and bone during prenatal and infantile growth periods	Japanese	overexpressed in DDH OA	(18)
chromosome 4q34-35	teneurin transmembrane protein 3 (*TENM3)*	teneurin transmembrane protein 3	multifunction transmembrane protein involved in signal transduction in developing limb and expressed in prechondrogenic mesenchymal cells	Caucasian	mutation discovered in a multigeneration DDH-affected family	(20)
chromosome 4q35	C-X3-C motif chemokine receptor 1 (*CX3CR1)*	C-X3-C motif chemokine receptor 1	mediates cellular adhesive and migratory functions and is expressed in MSCs destined to become chondrocytes	Caucasian	mutation with a deleterious effect found in 4 generations, 72 family members affected by DDH	(21)
chromosome 6q25	estrogen receptor 1 (*ER1)*	Estrogen receptor 1	regulates transcriptional responses	Caucasian	possible correlation of gene polymorphism to DDH	(22)
chromosome 9q22	asporin *(ASPN)*	asporin	extracellular matrix protein that can bind to TGF-β1 and sequentially inhibit TGF-β/Smad signaling	Han Chinese	aspartic acid repeat polymorphism is an important regulator in the etiology of DDH	(23)
chromosome 12q	vitamin D receptor *(VDR)*	vitamin D receptor	regulates transcriptional responses	Caucasian	possible correlation of gene polymorphism to DDH	(22)
				Caucasian	possible link between some haplotypes and the risk of severe OA in patients with DDH	(24)
	collagen type II alpha I chain *(COL2A1)*	collagen type II alpha I chain	involved in collagen type II synthesis	Caucasian	possible link between some haplotypes and the risk of severe OA in patients with DDH	(24)
chromosome 13q22	*/*	/	/	Japanese	autosomal dominant inheritance with a considerably consistent phenotype	(25)
chromosome 17q21	homebox (*HOX)* genes cluster	/	developmental regulatory system providing cells with specific positional identities along the developing joint and spine	/	possible linkage to DDH	(26)
				Chinese	associated with DDH	(27)
	homebox B9 *(HOXB9)*	homeobox B9	involved in embryonic limb developments	Chinese	may be susceptibility gene	(27)
	collagen type II alpha I chain *(COL1A1)*	collagen type II alpha I chain	involved in collagen type I synthesis	Chinese	may be susceptibility gene for DDH	(27)
chromosome 17q21-q2	pregnancy-associated plasma protein-A2 (*PAPPA2)*	pregnancy-associated plasma protein-A2	regulates IGF release and effect, important for the development of the fetus and normal postnatal growth	Han Chinese	single SNP associated with sporadic DDH	(28)
chromosome 17q23	T-box transcription factor 4 (*TBX4)*	T-box transcription factor 4	transcription factor involved in formation of posterior mesoderm and axial development	Han Chinese	one SNP associated with DDH in both genders, one SNP only in male patients	(29)
chromosome 19p13	Krüppel-like Factor 2 *(KLF2)*	Krüppel-like Factor 2	potential regulator of expression of MMPs	Japanese	overexpressed in DDH OA	(18)
chromosome 20q11	growth differentiation factor 5 *(GDF5)*	growth differentiation factor 5	a member of TGF- β superfamily, closely related to BMP subfamily, key role in osteogenesis, chondrogenesis and joint formation	Han Chinese	single functional SNP associated with DDH	(30)
				Han Chinese	two SNPs associated with DDH in female population	(31)
				Caucasian	two SNPs associated with DDH in Caucasian population	(17)
	*GDF5* promotor			Iranian	hypermethylated in cartilage samples, indicating a potential role in DDH development	(32)

*MSCs – mesenchymal stem cells; OA – osteoarthritis; ATP – adenosine triphosphate; TGF – transforming growth factor; IGF – insulin-like growth factor; SNP – single nucleotide polymorphism; MMP – matrix metalloproteinase; BMP – bone morphogenetic proteins.

**Table 2:** Studies of genetic background of developmental dysplasia of the hip (DDH) showing no correlation between investigated genes and the disease in different populations*

Locus	Gene	Protein	Function	Population	Correlation to DDH	Reference
chromosome 12q	collagen type II alpha I chain *(COL2A1)*	collagen type II alpha I chain	involved in collagen type II synthesis	Caucasian	although variants in these genes may contribute to the development of OA in DDH patients, they do not contribute to DDH	(33)
	vitamin D receptor *(VDR)*	vitamin D receptor	regulates transcriptional responses	Caucasian		
				Saudi Arabian	no significant association between 4 SNPs and DDH	(34)
chromosome 17q21	collagen type I alpha I chain *(COL1A1)*	collagen type I alpha I chain	involved in collagen type I synthesis	Chinese	polymorphisms of PCOL2 (in promoter) and Sp1 (in intron 1) binding sites in *COL1A1* gene may not be the major susceptibility genes of DDH	(35)
				Caucasian	no significant association between 8 SNPs and DDH	(36)
	homeobox B9 (*HOXB9)*	homeobox B9	involved in embryonic limb developments	Caucasian	no significant association between 2 SNPs and DDH	(36)

*OA – osteoarthritis; SNP – single nucleotide polymorphism.

A recent study, performed on samples taken from the external portion of the femoral head-neck junction, assessed the association between DDH and metabolic disorders of the cartilage in DDH patients, patients with primary OA, and patients with femoral neck fracture (FNF). DDH patients had lower mRNA expression of collagen type II and aggrecan compared with other groups. Furthermore, they had higher mRNA expression of collagen type X and matrix metalloproteinase-13 (*MMP-13*), as cartilage degradation markers, than the FNF group, but the expression did not differ from the OA group. The authors concluded that the DDH group had more impaired cartilage metabolism than the OA group. MMP-13 is responsible for the degradation of collagen type II and other collagens (37) and MMP-13 in human chondrocytes was reported to be decreased by growth differentiation factor-5 (GDF-5) (38), which might explain a part of the DDH cascade.

Aki et al (18) compared the whole-genome chondrocyte transcriptomics in Japanese patients with DDH-associated secondary OA and FNF with that of Caucasian patients suffering from primary hip OA, whose data were available in the literature. The cartilage specimens of patients with secondary OA were obtained from a location on the femoral head adjacent to the weight-bearing area, while the specimens of FNF patients were obtained from the inner and intact, non-weight bearing area of the femoral head. The study found 888 up-regulated and 732 down-regulated genes in DDH-induced secondary OA patients in comparison with FNF patients. The authors also reported that 90% of up-regulated genes in primary OA were different from those in DDH-induced secondary OA. Furthermore, DDH-induced secondary OA samples had high dermatopontin (*DPT*), insulin-like growth factor-binding protein 7 (*IGFBP7*), and Krüppel-like factor 2 (*KLF2*) expression (18). DPT is a protein of the extracellular matrix (ECM) that interacts with transforming growth factor β1 (TGF-β1) and decorin, inhibiting the formation of decorin-TGF-β1 complex, thus enhancing the effect of TGF-β1 (39). TGF-β1 plays a role in bone remodeling, parenchymal fibrosis, blood vessel formation, fetal growth, wound healing, and other pleiotropic roles in prenatal development and postnatal life (known role in cancer, inflammation, etc) (18,40,41). IGFBPs are a part of the insulin growth factor-1 (IGF-1) metabolic pathway (42). IGF-1 induces mesenchymal to osteoblast differentiation and is highly expressed in the bone matrix, having a crucial role in bone formation (43). It circulates in the blood and then binds to IGFBPs, which control the distribution and activity of the IGF-1 with its receptors. IGFBP7 suppresses osteoclastogenesis and supports osteogenic differentiation of bone marrow-derived mesenchymal stem cells (42). IGFBPs are processed by the bone morphogenetic protein-1 (BMP-1) metalloproteinase, which also releases BMP-2 and BMP-4 from their latent complexes (44). Both of these molecules play an essential role in osteogenesis and chondrogenesis and could be important in DDH development (45). KLF2 inhibits macrophage and endothelial activation in inflammation, as well as regulates cartilage degeneration by inhibiting *MMP-13* expression, thus regulating the cleavage of collagen type II (18,46). Surprisingly, Aki et al showed an up-regulation of *KLF2* expression in secondary DDH-associated OA, while previous studies showed *KLF2* down-regulation in OA patients (18,46). The authors contribute this difference to a different OA stage in their patients (18). However, in our opinion, this finding needs further clarification.

## HISTOLOGY OF THE ARTICULAR CARTILAGE IN DDH

**Figure 1:**
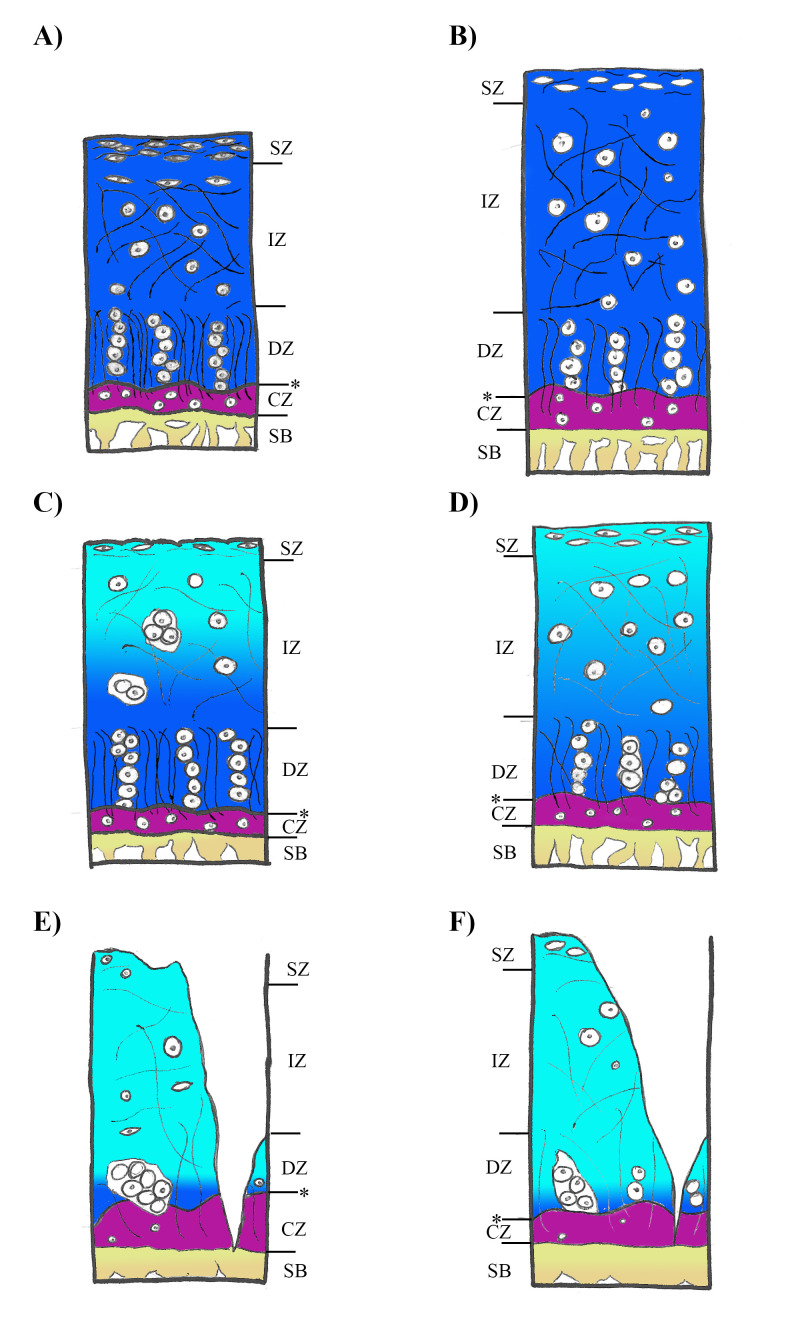
Normal articular cartilage and articular cartilage in developmental dysplasia of the hip (DDH) at different stages of osteoarthritis (OA). (**A**) Normal articular cartilage. The cartilage surface is smooth, chondrocytes and collagenous fibrils are organized into the superficial, middle, and deep zone. A tidemark clearly separates the deep from the calcified zone. Proteoglycans are present in all three cartilage zones. The subchondral bone plate and the trabeculae are thin. (**B**) Non-OA DDH. The cartilage surface is smooth, cartilage thickness is increased, and chondrocyte count is high. Collagenous fibrils are rare compared with normal cartilage. (**C**) Early-stage primary OA. The cartilage surface is intact, but superficial fibrillation is visible at some regions of the surface. Chondrocytes and collagenous fibrils are organized, but are fewer in number. Non-viable cells are visible, especially in the superficial zone. The color fading in the upper third of the cartilage indicates the loss of proteoglycans. Bone trabeculae are thicker. (**D**) Early-stage DDH-induced secondary OA. The cartilage surface is smooth. Total cell count is unchanged, but the incidence of cell death is high, especially in the superficial zone. Collagenous fibrils are rare in comparison with normal cartilage. Some proteoglycan loss is visible at the upper third of the cartilage. (**E**) Late-stage primary OA. The cartilage surface is eroded and fibrillated. The chondrocyte count and the number of viable cells are significantly decreased. Chondrocytes form aggregates, while collagenous fibrils are rare and disorganized. The tidemark area is significantly increased. The loss of color indicates a decrease in total proteoglycan content. The subchondral bone plate and trabeculae are thicker. (**F**) Late-stage DDH-induced secondary OA. The cartilage surface is fibrillated and eroded. There are a few viable chondrocytes, which are shrunken at some places and enlarged at others. Chondrocytes are irregularly organized, aggregating in some regions, while being absent in others. Collagenous fibrils are rare and without any structure. A significant loss of proteoglycans is present. SZ – superficial zone; IZ – intermediate (middle) zone; DZ – deep zone; asterisk – tidemark; CZ – calcified zone; SB – subchondral zone.

The DDH-affected acetabular and femoral cartilage show similar histological changes. These changes are most noticeable in the iliac acetabular growth plate, where irregularly arranged chondrocytes are observed, shrunken at some places and enlarged at others, showing a tendency for degeneration. In the femoral head, dysmorphic chondrocytes are visible, which aggregate in some areas and are absent in others (47,48). In DDH-affected hips, the acetabular articular cartilage is thicker than in healthy hips (Figure 1). This phenomenon can be observed already during infancy and is used as a sign in the early diagnosis of DDH (49,50). Cartilage collagenous fibrils in DDH are rare and disorganized, similar to decreased and scattered fibrils in OA patients.

Histological analysis has shown that the chondrocyte count in the articular cartilage is significantly decreased in primary OA, while, interestingly, it is not affected in DDH (37). Some characteristics of DDH-induced secondary OA are not typical for primary OA. In primary OA, the weight-bearing regions of the femur exhibit a higher degree of cartilage degradation, while the cartilage in the non-weight bearing regions is usually still healthy or shows only early signs of degenerative changes. However, in DDH, the non-weight bearing region also shows various degrees of erosion. Early-stage OA in DDH patients is characterized by fully thick cartilage with a smooth surface and insignificant loss of proteoglycans. The chondrocyte density is high, but so is the occurrence of cell death, especially in the superficial zone – which is not typically observed in the early stages of primary OA. The remaining cells are metabolically highly active. As shown by immunostaining for proteases, a vast majority of viable chondrocytes produce a proteolytic enzyme (ie, MMP-3 or MMP-9), thus contributing to the matrix and cartilage degradation. More advanced OA stages in DDH patients are accompanied by a more significant proteoglycan loss, especially in the surface regions. The cartilage surface is fibrillated with a small number of viable cells, which produce proteases (MMP-3, MMP-9, MMP-13, ADAMTS-4) (51).

OA changes are associated with the changes in the abundance of major ECM macromolecules, primarily collagen type II, aggrecan, and cartilage degradation markers (52). The content of collagen type II decreases with the progression of cartilage degradation, meaning that primary OA-affected cartilage has lower collagen type II levels than healthy cartilage. As expected, DDH-induced secondary OA cartilage also has decreased collagen type II content, which is even more pronounced in comparison with the primary OA group. Another indicator of cartilage degradation are decreased aggrecan levels in the cartilage matrix, which are lower in DDH-induced secondary OA than in healthy cartilage and primary OA-affected cartilage (37).

The most important cartilage degradation markers are collagen type X, MMPs, and keratan sulfate (KS) (37,53,54). Collagen type X is particularly expressed in hypertrophic chondrocytes and constitutes approximately 1% of the total amount of collagen in normal cartilage. Its expression is increased in degenerative joint diseases, such as OA (55). Collagen type X content is significantly higher in DDH-induced secondary OA tissue than in healthy cartilage and is similar to that in primary OA cartilage (37).

MMPs are the enzymes responsible for most of the proteolytic reactions associated with OA, with low expression in healthy cartilage. Based on their substrate specificity, they are divided into several groups, the most important being collagenases, disintegrin, and metalloproteinases with thrombospondin motifs (ADAMTSs) (52). Their levels increase with advanced degradation caused by either primary or DDH-induced secondary OA, and this increase explains the loss of collagen type II in DDH (37). So far, there has been little information on the difference between DDH-induced and primary OA cartilage tissue in MMP protein content. Some studies on DNA methylation and gene silencing have confirmed the differences in *MMP-13* expression levels between the conditions, indicating a possible difference in total MMP-13 protein content (51). However, other studies found no such difference (37). Due to the scarcity of these studies, the results are inconclusive and further research is warranted.

ADAMTSs are a group of metabolically active metalloproteinases involved in various developmental and homeostatic processes (ie, fertilization, cartilage metabolism, intravascular coagulation, von Willebrand factor activation) (56). Nineteen ADAMTSs enzymes have been described (57). Some of them play a crucial role in cartilage degradation as they act as aggrecanases, characterized by enzymatic cleavage at the Glu373-Ala374 peptide bond of the aggrecan (56). The first two described aggrecanases are ADAMTS-4 and ADAMTS-5. ADAMTS-4 is induced as a response to numerous inflammatory cytokines, ie, TGF-β, TNF-α, and IL-1β, while ADAMTS-5 is found in human cartilage in homeostasis and is essential for balancing between aggrecan catabolism and anabolism (58,59). Their expression profiles have so far been studied in primary OA, with an increased expression indicating degenerative processes and correlating with aggrecan loss and disease progression (59). However, it is still unclear whether the altered *ADAMTSs* expression is directly responsible for the disease pathogenesis or it is only a consequence of homeostasis imbalance (45). As with the other cartilage degradation markers, there is limited information on *ADAMTSs* expression in DDH-induced secondary OA tissue, but the same ADAMTS-4 content has been found in both DDH-induced and primary OA tissue (51). Moreover, ADAMTS-4 can degrade other constitutive molecules in the ECM of the human cartilage, such as cartilage oligomeric matrix protein, hyalectan, decorin, brevican, fibromodulin, etc. Therefore, ADAMTS-4 shows a broader proteolytic role in the metabolism, which should be addressed in terms of involvement in DDH-induced secondary OA pathogenesis (60).

Some of the other noteworthy biomarkers are MMP-3 and KS. Even though both MMP-3 and KS serve as markers of cartilage degradation, and their expression increases with arthritic changes, there is no notable difference in their levels in early and advanced stages of DDH-induced secondary OA (53).

## RADIOLOGICAL CHARACTERISTICS OF THE ARTICULAR CARTILAGE IN DDH

An ultrasound study showed an average acetabular cartilage thickness of 2.6 mm in healthy infants and of 4.6 mm in infants with DDH (49). A magnetic resonance imaging (MRI) study also reported that dysplastic hips cartilage was thicker than that of normal hips (1.77 mm vs 1.34 mm), which correlates with histological findings (61). Both groups showed an increased thickness in the superior lateral area, but in dysplastic hips the increase was significant (61). Another study reported that dysplastic hips without cartilage wear had relatively thicker acetabular cartilage in the anterosuperior area than healthy hips, while both groups had similar femoral cartilage thickness (62). In dysplastic hips, the acetabular edge was thickened, being either inverted or everted (63).

In 88 children with unilateral DDH, acetabular depth (AD) in the affected hip was significantly smaller compared with that in the contralateral unaffected hip. Also, with increased age, AD increase on the affected side was significantly smaller compared with that on the contralateral side (64). In children younger than three years with untreated DDH, the floor of the articular portion of the acetabulum advanced laterally, as evidenced by the thickening of the bony acetabulum portion immediately superior to triradiate cartilage (63). Also, the bony acetabulum lost sphericity in the anterior, superior, and posterior parts. Anterior and posterior acetabular rims were rotated toward each other, producing a reduced acetabular diameter (63).

Lateral acetabular coverage correlates with femoroacetabular cartilage thickness, which indicates that the cartilage may transform under certain circumstances. In dysplastic hips, the cartilage at the lateral part was 35% thicker compared with the hips with normal acetabular coverage, suggesting that the hip morphologically changes in response to abnormal joint loading (65). One study showed a more prominent T2 signal decrease in the outer superficial acetabular cartilage of dysplastic hips during weight loading in comparison with normal hips. This finding may be explained by changes in ECM and water distribution caused by abnormal weight bearing forces in DDH patients (66). In addition, we assume that these changes correlate with the histological evidence of lower proteoglycans content in DDH articular cartilage in comparison with healthy articular cartilage, which indirectly alters the water content in the cartilage.

Jessel et al (67) compared 96 symptomatic dysplastic hips of 74 DDH patients with the hips of healthy volunteers. The mean delayed gadolinium-enhanced MRI of cartilage (dGEMRIC) index for DDH patients with hip symptoms was 473 ± 104 msec, while the value for healthy volunteers was 570 ± 90 msec, suggesting a decrease in proteoglycans in symptomatic pre-OA DDH patients (67). Another study correlated dGEMRIC index of the weight bearing cartilage in DDH patients with dysplasia severity and pain, suggesting that dGEMRIC index might indicate early OA before radiographic changes are present (68). Arthroscopic and imaging studies showed that cartilage erosions in pre-OA DDH patients are usually located in the anterior superior part of the acetabulum, most commonly in the transitional zone between the cartilage and labrum (69-71).

In the early stages of DDH-induced secondary OA, T2 signal intensity in the superior acetabular cartilage was higher in comparison with normal hips, probably as a result of increased fluid amount and mobility, as well as of a loss of proteoglycans and disintegration of collagen network (72). DDH patients with total dGEMRIC index above 500 msec, ie, pre-OA DDH patients, had increased T1 values in the weight bearing area of the acetabulum, with lower values in peripheral regions compared with central regions. Patients with total dGEMRIC index below 500 msec, ie, DDH-induced secondary OA patients, had generally decreased T1 values, with no significant difference between central and peripheral regions (73).

In secondary DDH-associated OA, the cartilage thinned with disease progression. Due to the cartilage thinning, in approximately 80% of patients, a transition of the femoral head to an anterior and superior position was observed (74). Xu et al (75) studied dGEMRIC indices in different stages of DDH-induced secondary OA. Mild radiographic DDH-induced secondary OA patients showed a decline in chondral function from anterior-superior toward the superior region, while moderate and severe radiographic DDH-induced secondary OA patients exhibited global chondral dysfunction (75).

Delayed gadolinium-enhanced MRI of cartilage index values of the cartilage surrounding focal chondral erosions were lower in DDH hips than in the normal cartilage, suggesting that the chondral erosions occur in combination with a global loss of proteoglycans. This indicates that DDH patients suffer not localized, but generalized, chondral damage (76). This observation may be related to the finding that DDH hips have more metabolically active chondrocytes that produce enzymes causing cartilage degradation. Furthermore, Hingsammer et al (77) state that patients with early radiographic OA may have localized chondral lesions, while biochemical changes (measured by dGEMRIC index) of the cartilage are present in the joint, although mechanical forces affecting the cartilage are asymmetric and localized. According to the authors, these data suggest that OA development in DDH may be induced by mechanical forces; however, there is certainly a biologic factor involved (77).

## CONCLUSION

The current knowledge on DDH is comprehensive in terms of early screening and treatment. However, data on etiology and morphology behind DDH and DDH-induced secondary OA are scarce. In this review, we highlighted some of the molecules with a potentially crucial role in DDH and DDH-induced secondary OA pathogenesis that merit further investigation. In addition, as some genetically pre-determined articular surfaces do not articulate in DDH, while surfaces without a genetic predisposition articulate, we believe the hip joint requires further regional morphological characterization.
